# Supplementary material for: Circulating tumour mutation detection in triple-negative breast cancer as an adjunct to tissue response assessment
Source: NPJ Breast Cancer. 2024 Jan 5;10:3. doi: 10.1038/s41523-023-00607-1 (PMC10770342; doi:10.1038/s41523-023-00607-1)
Supplement: Supplementary file 1 — Supplementary Information [file 41523_2023_607_MOESM1_ESM.pdf]

Supplementary Table 1. Probes used for mutation validation using ddPCR. Probes were purchased from BioRad and the custom oligos were ordered through IDT using sequences available at BioRad for each specific probe.

Target gene	Variant	Assay ID	Probe Fluorophore	Positive control
**TP53**	**R213***	dHSACP2506810	FAM (Mut)	Custom oligo
		dHSACP2506811	HEX (WT)	
	**A161T**	dHSACP2506786	FAM (Mut)	
		dHSACP2506787	HEX (WT)	
	**G266E**	dHSACP2506854	FAM (Mut)	
		dHSACP2506855	HEX (WT)	
	**R273C**	dHSACP2500538	FAM (Mut)	
		dHSACP2500539	HEX (WT)	
	**R175H**	dHSACP200105	FAM (Mut)	
		dHSACP200106	HEX (WT)	
	**C238Y**	dHSACP2506850	FAM (Mut)	
		dHSACP2506851	HEX (WT)	
	**I195T**	dHSACP2506898	FAM (Mut)	
		dHSACP2506899	HEX (WT)	
	**R306***	dHSACP2500552	FAM (Mut)	
		dHSACP2500553	HEX (WT)	
	**R273H**	dHSACP200109	FAM (Mut)	
		dHSACP200110	HEX (WT)	
**KRAS**	**G12S**	dHSACP2500588	FAM (Mut)	Custom oligo or Horizon HD780 multiplex ctDNA reference standard
		dHSACP2500589	HEX (WT)	
	**G12A**	dHSACP2500586	FAM (Mut)	
		dHSACP2500587	HEX (WT)	
	**G12V**	dHSACP2500592	FAM (Mut)	
		dHSACP2500593	HEX (WT)	
	**G12D**	dHSACP2500596	FAM (Mut)	
		dHSACP2500597	HEX (WT)	
	**G12C**	dHSACP2500584	FAM (Mut)	Custom oligo
		dHSACP2500585	HEX (WT)	

PIK3CA	E545K	dHSACP2000075	FAM (Mut)	MCF-7 cell line or Horizon HD780 multiplex ctDNA reference standard
		dHSACP2000076	HEX (WT)	
	E542K	dHSACP2000073	FAM (Mut)	Custom oligo
		dHSACP2000074	HEX (WT)	
	H1047R	dHSACP2000077	FAM (Mut)	HCT116 cell line
		dHSACP2000078	HEX (WT)	
IDH1	R132H	dHSACP200055	FAM (Mut)	Custom oligo
		dHSACP200056	HEX (WT)	
	R132G	dHsaMDV2510512	FAM/HEX (Mut/WT)	Custom oligo
NRAS	Q61R	dHSACP200071	FAM (Mut)	Custom oligo
		dHSACP200072	HEX (WT)	
BRAF	V600E	dHSACP200027	FAM (Mut)	Custom oligo
		dHSACP200028	HEX (WT)	
POLE	T457T	dHsaMDS230604489	FAM/HEX (Mut/WT)	Custom oligo
	T457M	dHsaMDS195642452	FAM/HEX (Mut/WT)	Custom oligo
EGFR	A722V	dHsaMDS839693386	FAM/HEX (Mut/WT)	Custom oligo

Supplementary Table 2. Clinical characteristics of participants in cohort, and their comparison between NAT and ADJ participants, indicated by p-values for chi-square (categorical variables) and Wilcoxon tests (continuous variables). A *p*-value < 0.05 was considered statistically significant.

		All (n=130)	NAT (n=64)	Adjuvant (n=66)	*p*-value
Age, in years, at diagnosis range (median)		24 - 78 (54)	24 - 76 (52)	32 - 78 (56)	0.08
Node status (%)	Positive	53 (40.8)	35 (54.7)	18 (27.3)	0.0009
	Negative	75 (57.7)	27 (42.2)	48 (72.7)	
	Unknown	2 (1.5)	2 (3.1)	-	
LVI (%)	Present	20 (15.4)	6 (9.4)	14 (21.2)	0.01
	Not identified	97 (74.6)	48 (75.0)	49 (74.2)	
	Suspicious	3 (2.3)	1 (1.6)	2 (3.0)	
	Indeterminate	1 (0.8)	-	1 (1.5)	
	Unknown	9 (6.9)	9 (14.1)	-	
Stage (%)	T1	38 (29.2)	10 (15.6)	28 (42.4)	8e-05
	T2	65 (50.0)	32 (50.0)	33 (50.0)	
	T3	22 (16.9)	17 (26.6)	5 (7.6)	
	T4	5 (3.9)	5 (7.8)	-	
Grade (%)	2	27 (20.8)	14 (21.9)	13 (19.7)	0.9
	3	102 (78.5)	50 (78.1)	52 (78.8)	
	Unknown	1 (0.8)	-	1 (1.5)	
Tumour size, in cm, range (median)		0.25 - 9.30 (2.6)	0.25 - 8.00 (3.4)	0.70 - 9.30 (2.2)	9e-06
Clinical genetics (%)	Negative	56 (43.1)	29 (45.3)	27 (40.9)	0.9
	Variant identified	27 (20.8)	14 (21.9)	13 (19.7)	
	VUS	20 (15.4)	9 (14.1)	11 (16.7)	
	Not done	27 (20.8)	12 (18.8)	15 (22.7)	
Median interval between treatment end and post-treatment sample (months)		1.8	1.7	2.4	0.6
Recurrence or death (%)		17 (13.1)	10 (15.6)	7 (10.6)	0.4
Median interval between treatment end and recurrence or death (months)		10.8	6.8	16.3	0.2
Treatment type (%)	Triple therapy, anthracycline and alkylating, then taxane	68 (52.3)	26 (40.6)	42 (63.6)	1e-09

	Dual therapy	20 (15.4)	2 (3.1)	18 (27.3)	
	Triple therapy, taxane first, then anthracycline and alkylating	31 (23.8)	27 (42.2)	4 (6.1)	
	Protocol contains platinum	10 (7.7)	9 (14.1)	1 (1.5)	
	Other	1 (0.8)	-	1 (1.5)	
Radiation (%)	Yes	113 (86.9)	60 (93.8)	53 (80.3)	0.05
	No	16 (12.3)	4 (6.2)	12 (18.1)	
	Other	1 (0.8)	-	1 (1.5)	
Pathologic complete response in NAT participants (%)	Yes	-	24 (37.5)	-	-
	No	-	40 (62.5)	-	

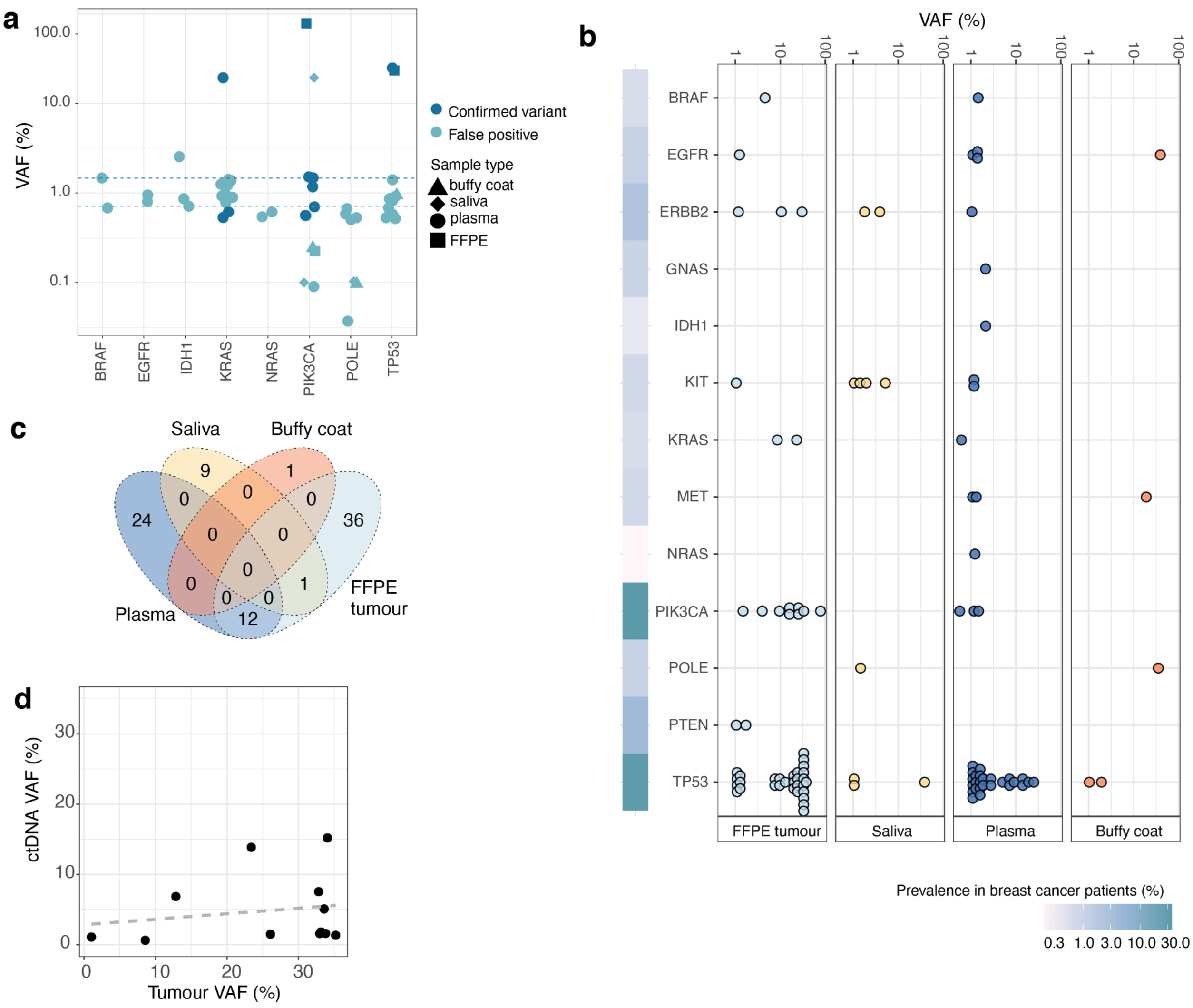

Supplementary Figure 1.(**a**) Distribution of VAF (%) across genes for variants validated by digital droplet PCR (ddPCR). The dashed lines represent the median VAF (%) for confirmed variants (dark blue) and false positive variants (light blue). (**b**) Comparison of VAF (%) in genes containing detectable mutations by sample type; breast cancer patient population (METABRIC and TCGA) prevalence of mutations in these genes are indicated by the heatmap. (**c**) Participant-specific overlap of variants in different sample types to identify likely germline variants and evaluate clonal hematopoiesis. (**d**) Correlation of VAFs for variants identified in participant-matched tumour and plasma samples.

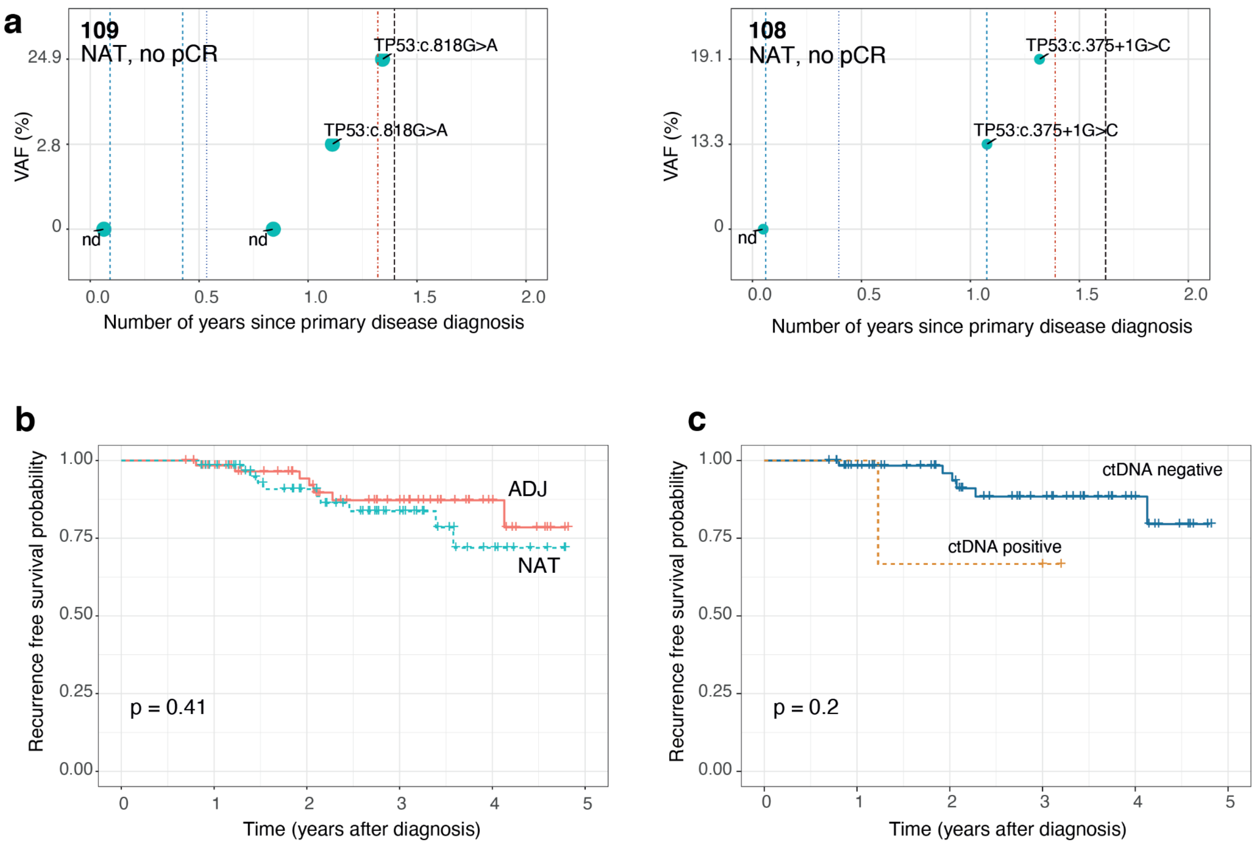

Supplementary Figure 2. (**a**) Variants detected in serial plasma samples from 2 NAT participants with an incomplete response. Circles represent targeted panel-sequenced plasma samples, red dashed line represents recurrence diagnosis, blue dashed lines are the first and last chemo treatments, and the purple dotted line indicates date of surgical resection. (**b**) Recurrence-free survival in participants in cohort, faceted by NAT or ADJ chemotherapy, and (**c**) recurrence-free survival in participants receiving ADJ chemotherapy, faceted by ctDNA status.

Supplementary Table 3. Somatic variants detected in plasma and FFPE tumour samples.

Gene name	RefSeq ID	cDNA change	Strand	Mutation type	Predicted impact (SnpEff)
BRAF	NM_004333.4	c.1397G>T	-1	missense_variant	MODERATE
BRAF	NM_004333.4	c.1780G>A	-1	missense_variant	MODERATE
EGFR	NM_005228.3	c.2139A>G	1	synonymous_variant	LOW
EGFR	NM_005228.3	c.2327G>A	1	missense_variant	MODERATE
EGFR	NM_005228.3	c.2458C>T	1	stop_gained	HIGH
EGFR	NM_005228.3	c.2611G>A	1	missense_variant	MODERATE
ERBB2	NM_004448.3	c.2329G>T	1	missense_variant	MODERATE

ERBB2	NM_004448.3	c.2463C>T	1	synonymous_variant	LOW
ERBB2	NM_004448.3	c.2464C>T	1	synonymous_variant	LOW
ERBB2	NM_004448.3	c.929C>T	1	missense_variant	MODERATE
GNAS	NM_080425.3	c.2531G>A	1	missense_variant	MODERATE
IDH1	NM_001282386.1	c.394C>G	-1	missense_variant	MODERATE
KIT	NM_000222.2	c.1497G>A	1	synonymous_variant	LOW
KIT	NM_000222.2	c.1753C>T	1	missense_variant	MODERATE
KRAS	NM_033360.3	c.35G>A	-1	missense_variant	MODERATE
KRAS	NM_033360.3	c.35G>T	-1	missense_variant	MODERATE
MET	NM_001127500.2	c.2847C>T	1	synonymous_variant	LOW
MET	NM_001127500.2	c.2883A>G	1	synonymous_variant	LOW
NRAS	NM_002524.4	c.182A>G	-1	missense_variant	MODERATE
PIK3CA	NM_006218.3	c.1624G>A	1	missense_variant	MODERATE
PIK3CA	NM_006218.3	c.1633G>A	1	missense_variant	MODERATE
PIK3CA	NM_006218.3	c.3140A>G	1	missense_variant	MODERATE
PTEN	NM_001304717.2	c.908G>A	1	missense_variant	MODERATE
TP53	NM_000546.5	c.105G>A	-1	synonymous_variant	LOW
TP53	NM_000546.5	c.167A>G	-1	missense_variant	MODERATE
TP53	NM_000546.5	c.181G>A	-1	missense_variant	MODERATE
TP53	NM_000546.5	c.212C>A	-1	missense_variant	MODERATE
TP53	NM_000546.5	c.242C>T	-1	missense_variant	MODERATE
TP53	NM_000546.5	c.309C>T	-1	synonymous_variant	LOW
TP53	NM_000546.5	c.331C>T	-1	synonymous_variant	LOW
TP53	NM_000546.5	c.332T>C	-1	missense_variant	MODERATE
TP53	NM_000546.5	c.374C>T	-1	missense_variant&splice_region_variant	MODERATE
TP53	NM_000546.5	c.375+1G>C	-1	splice_donor_variant&intron_variant	HIGH
TP53	NM_000546.5	c.378C>T	-1	splice_region_variant&synonymous_variant	LOW
TP53	NM_000546.5	c.413C>T	-1	missense_variant	MODERATE
TP53	NM_000546.5	c.476C>T	-1	missense_variant	MODERATE
TP53	NM_000546.5	c.488A>G	-1	missense_variant	MODERATE
TP53	NM_000546.5	c.527G>A	-1	missense_variant	MODERATE

TP53	NM_000546.5	c.584T>C	-1	missense_variant	MODERATE
TP53	NM_000546.5	c.587G>A	-1	missense_variant	MODERATE
TP53	NM_000546.5	c.604C>T	-1	missense_variant	MODERATE
TP53	NM_000546.5	c.614A>G	-1	missense_variant	MODERATE
TP53	NM_000546.5	c.637C>T	-1	stop_gained	HIGH
TP53	NM_000546.5	c.639A>G	-1	synonymous_variant	LOW
TP53	NM_000546.5	c.641A>G	-1	missense_variant	MODERATE
TP53	NM_000546.5	c.646G>A	-1	missense_variant	MODERATE
TP53	NM_000546.5	c.713G>A	-1	missense_variant	MODERATE
TP53	NM_000546.5	c.722C>T	-1	missense_variant	MODERATE
TP53	NM_000546.5	c.743G>A	-1	missense_variant	MODERATE
TP53	NM_000546.5	c.782+1G>A	-1	splice_donor_variant&intron_variant	HIGH
TP53	NM_000546.5	c.782+2T>G	-1	splice_donor_variant&intron_variant	HIGH
TP53	NM_000546.5	c.801G>C	-1	synonymous_variant	LOW
TP53	NM_000546.5	c.813G>A	-1	synonymous_variant	LOW
TP53	NM_000546.5	c.816G>C	-1	synonymous_variant	LOW
TP53	NM_000546.5	c.817C>T	-1	missense_variant	MODERATE
TP53	NM_000546.5	c.818G>A	-1	missense_variant	MODERATE
TP53	NM_000546.5	c.818G>T	-1	missense_variant	MODERATE
TP53	NM_000546.5	c.819T>A	-1	synonymous_variant	LOW
TP53	NM_000546.5	c.825T>C	-1	synonymous_variant	LOW
TP53	NM_000546.5	c.832C>A	-1	missense_variant	MODERATE
TP53	NM_000546.5	c.839G>A	-1	missense_variant	MODERATE
TP53	NM_000546.5	c.841G>C	-1	missense_variant	MODERATE
TP53	NM_000546.5	c.843C>A	-1	missense_variant	MODERATE
