# Supplementary material for: Circulating tumour mutation detection in triple-negative breast cancer as an adjunct to tissue response assessment
Source: NPJ Breast Cancer. 2024 Jan 5;10:3. doi: 10.1038/s41523-023-00607-1 (PMC10770342; doi:10.1038/s41523-023-00607-1)
Supplement: Supplementary file 2 — Reporting-summary [file 41523_2023_607_MOESM2_ESM.pdf]

nature portfolio

Corresponding author(s): Samuel Aparicio & Karen Gelmon

Last updated by author(s): Nov 27, 2023

Reporting Summary

Nature Portfolio wishes to improve the reproducibility of the work that we publish. This form provides structure for consistency and transparency in reporting. For further information on Nature Portfolio policies, see our Editorial Policies and the Editorial Policy Checklist.

Statistics

For all statistical analyses, confirm that the following items are present in the figure legend, table legend, main text, or Methods section.

n/a	Confirmed	
☐	☒	The exact sample size (*n*) for each experimental group/condition, given as a discrete number and unit of measurement
☐	☒	A statement on whether measurements were taken from distinct samples or whether the same sample was measured repeatedly
☐	☒	The statistical test(s) used AND whether they are one- or two-sided<br>*Only common tests should be described solely by name; describe more complex techniques in the Methods section.*
☐	☒	A description of all covariates tested
☒	☐	A description of any assumptions or corrections, such as tests of normality and adjustment for multiple comparisons
☐	☒	A full description of the statistical parameters including central tendency (e.g. means) or other basic estimates (e.g. regression coefficient) AND variation (e.g. standard deviation) or associated estimates of uncertainty (e.g. confidence intervals)
☐	☒	For null hypothesis testing, the test statistic (e.g. *F*, *t*, *r*) with confidence intervals, effect sizes, degrees of freedom and *P* value noted<br>*Give P values as exact values whenever suitable.*
☒	☐	For Bayesian analysis, information on the choice of priors and Markov chain Monte Carlo settings
☒	☐	For hierarchical and complex designs, identification of the appropriate level for tests and full reporting of outcomes
☒	☐	Estimates of effect sizes (e.g. Cohen's *d*, Pearson's *r*), indicating how they were calculated

Our web collection on statistics for biologists contains articles on many of the points above.

Software and code

Policy information about availability of computer code

Data collection	Mutation data for Breast Cancer patient data from the METABRIC study was accessed using cBioPortal (Cerami et al, 2012)
Data analysis	Proprietary quality assurance methods based on DNA sequence barcodes that were incorporated into the assay and the bioinformatics pipeline, were used to increase sensitivity of called mutations. Mutations were called with a supervised classification method that returned the random forest (RF) probability score of a variant belonging to the mutation class as opposed to the artifact class, with classification based on the alignment, sequence composition, and barcode information of the variant. Reads passing the quality control filters were then aligned to the hg19 reference genome. Identified variants were then annotated using SnpEff v 4.3 (Cingolani et al, 2012).<br>Data analyses, including chi-square and Wilcoxon tests and Kaplan-Meier survival probability, as well as data presentation were performed using R (version 3.6.1, https://cran.r-project.org/) and visualized using:<br>ggplot2 (Wickham, 2016)<br>UpSetR (Conway et al, 2017)<br>survminer (https://github.com/kassambara/survminer)<br>ggvenn (https://github.com/yanlinlin82/ggvenn)<br>cowplot (https://github.com/wilkelab/cowplot)<br>patchwork (https://github.com/thomasp85/patchwork)

For manuscripts utilizing custom algorithms or software that are central to the research but not yet described in published literature, software must be made available to editors and reviewers. We strongly encourage code deposition in a community repository (e.g. GitHub). See the Nature Portfolio guidelines for submitting code & software for further information.

Data

Policy information about availability of data

All manuscripts must include a data availability statement. This statement should provide the following information, where applicable:

- Accession codes, unique identifiers, or web links for publicly available datasets
- A description of any restrictions on data availability
- For clinical datasets or third party data, please ensure that the statement adheres to our policy

Raw data from targeted panel sequencing are available in EGA under EGAS00001006937. Detected somatic mutations are reported in Supplementary Table 3.

Research involving human participants, their data, or biological material

Policy information about studies with human participants or human data. See also policy information about sex, gender (identity/presentation), and sexual orientation and race, ethnicity and racism.

Reporting on sex and gender

Use the terms sex (biological attribute) and gender (shaped by social and cultural circumstances) carefully in order to avoid confusing both terms. Indicate if findings apply to only one sex or gender; describe whether sex and gender were considered in study design; whether sex and/or gender was determined based on self-reporting or assigned and methods used.
Provide in the source data disaggregated sex and gender data, where this information has been collected, and if consent has been obtained for sharing of individual-level data; provide overall numbers in this Reporting Summary. Please state if this information has not been collected.
Report sex- and gender-based analyses where performed, justify reasons for lack of sex- and gender-based analysis.

Reporting on race, ethnicity, or other socially relevant groupings

Please specify the socially constructed or socially relevant categorization variable(s) used in your manuscript and explain why they were used. Please note that such variables should not be used as proxies for other socially constructed/relevant variables (for example, race or ethnicity should not be used as a proxy for socioeconomic status).
Provide clear definitions of the relevant terms used, how they were provided (by the participants/respondents, the researchers, or third parties), and the method(s) used to classify people into the different categories (e.g. self-report, census or administrative data, social media data, etc.)
Please provide details about how you controlled for confounding variables in your analyses.

Population characteristics

Research staff trained on the protocol at each participating BC Cancer centre identified eligible TNBC participants and obtained informed consent. Eligibility criteria were as follows: (1) diagnosed with any stage of TNBC, (2) 18 years or older, (3) informed consent provided prior to any study procedures. Pregnant participants and those with a history of previous invasive cancer of DCIS were included.

Recruitment

Materials used for this study were obtained under the Precision Medicine for Breast Cancer Research program. The research study was explained to the participants in a private medical setting and sufficient time was given to explain the protocol and have their questions answered. Consent for use of surgical and core biopsies and clinical records data was obtained. All participants provided written informed consent to take part in the study, and the study design followed good clinical research practices outlined by the Office of Biobank Education and Research (OBER). The study was carried out in accordance with the Declaration of Helsinki.

Ethics oversight

The ethics committee/IRB of BC Cancer gave ethical approval for this work.

Note that full information on the approval of the study protocol must also be provided in the manuscript.

Field-specific reporting

Please select the one below that is the best fit for your research. If you are not sure, read the appropriate sections before making your selection.

☒ Life sciences ☐ Behavioural & social sciences ☐ Ecological, evolutionary & environmental sciences

For a reference copy of the document with all sections, see nature.com/documents/nr-reporting-summary-flat.pdf

Life sciences study design

All studies must disclose on these points even when the disclosure is negative.

Sample size

A sample-size calculation was not performed, and sample size was established based on the number of participants (n=130) with targeted-panel sequenced matched normal and available post-treatment plasma within 7 months of treatment completion.

Data exclusions

Study exclusion criteria included known blood disorders or a history of other solid or hematologic malignancy within the preceding 5 years(except for appropriately treated CIS of the cervix, Stage I uterine cancer and non-melanoma skin carcinoma.
Data was included only from participants who had a post-treatment sample within 7 months of last chemotherapy or surgery and prior to recurrence diagnosis. Data filtering included removing variants outside of the panel target regions were excluded from analyses, and those with a random forest score of <0.7 and VAF <1%, which was experimentally-determined by ddPCR. Variants confirmed by ddPCR were added

back into the pool of detected variants, and those with negative ddPCR results were excluded from downstream analyses. Likely germline variants and those resulting from clonal hematopoiesis were excluded from further analysis.

Replication	To ensure reproducibility of panel sequencing, 3 controls were used: a no template control, and normal control (NA01953, Coriell Cell Repositories) and a Quantitative Multiplex Formalin Compromised DNA reference (HD798, Horizon Discovery). ddPCR experiments were performed in duplicated for each sample where enough material was available.
Randomization	No randomization of samples was performed as samples were not allocated to different experimental groups.
Blinding	Blinding was not applicable as there were no experimental groups and no intervention was analyzed.

Reporting for specific materials, systems and methods

We require information from authors about some types of materials, experimental systems and methods used in many studies. Here, indicate whether each material, system or method listed is relevant to your study. If you are not sure if a list item applies to your research, read the appropriate section before selecting a response.

Materials & experimental systems

n/a	Involved in the study
☒	☐ Antibodies
☒	☐ Eukaryotic cell lines
☒	☐ Palaeontology and archaeology
☒	☐ Animals and other organisms
☒	☐ Clinical data
☒	☐ Dual use research of concern
☒	☐ Plants

Methods

n/a	Involved in the study
☒	☐ ChIP-seq
☒	☐ Flow cytometry
☒	☐ MRI-based neuroimaging

Plants

Seed stocks	*Report on the source of all seed stocks or other plant material used. If applicable, state the seed stock centre and catalogue number. If plant specimens were collected from the field, describe the collection location, date and sampling procedures.*
Novel plant genotypes	*Describe the methods by which all novel plant genotypes were produced. This includes those generated by transgenic approaches, gene editing, chemical/radiation-based mutagenesis and hybridization. For transgenic lines, describe the transformation method, the number of independent lines analyzed and the generation upon which experiments were performed. For gene-edited lines, describe the editor used, the endogenous sequence targeted for editing, the targeting guide RNA sequence (if applicable) and how the editor was applied.*
Authentication	*Describe any authentication procedures for each seed stock used or novel genotype generated. Describe any experiments used to assess the effect of a mutation and, where applicable, how potential secondary effects (e.g. second site T-DNA insertions, mosiacism, off-target gene editing) were examined.*
